# Supplementary material for: Genome-Wide Identification and Expression Analysis of Auxin Response Factor Gene Family in Linum usitatissimum
Source: Int J Mol Sci. 2023 Jul 2;24(13):11006. doi: 10.3390/ijms241311006 (PMC10341394; doi:10.3390/ijms241311006)
Supplement: Supplementary file 1 [file ijms-24-11006-s001.zip › Supplementary files/Supplementary Figures.pdf]

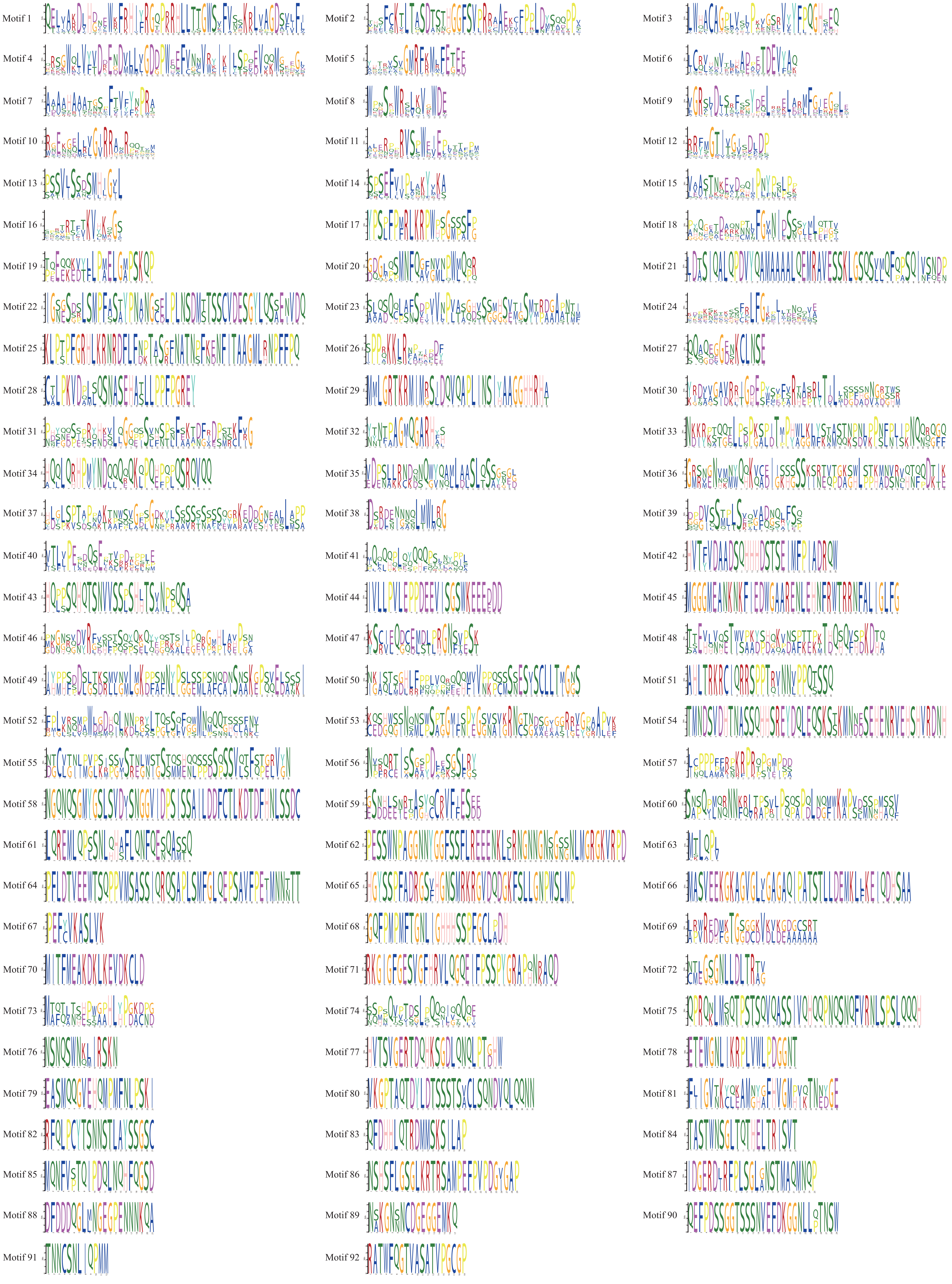

Figure S1. Conserved motifs of LuARF family.

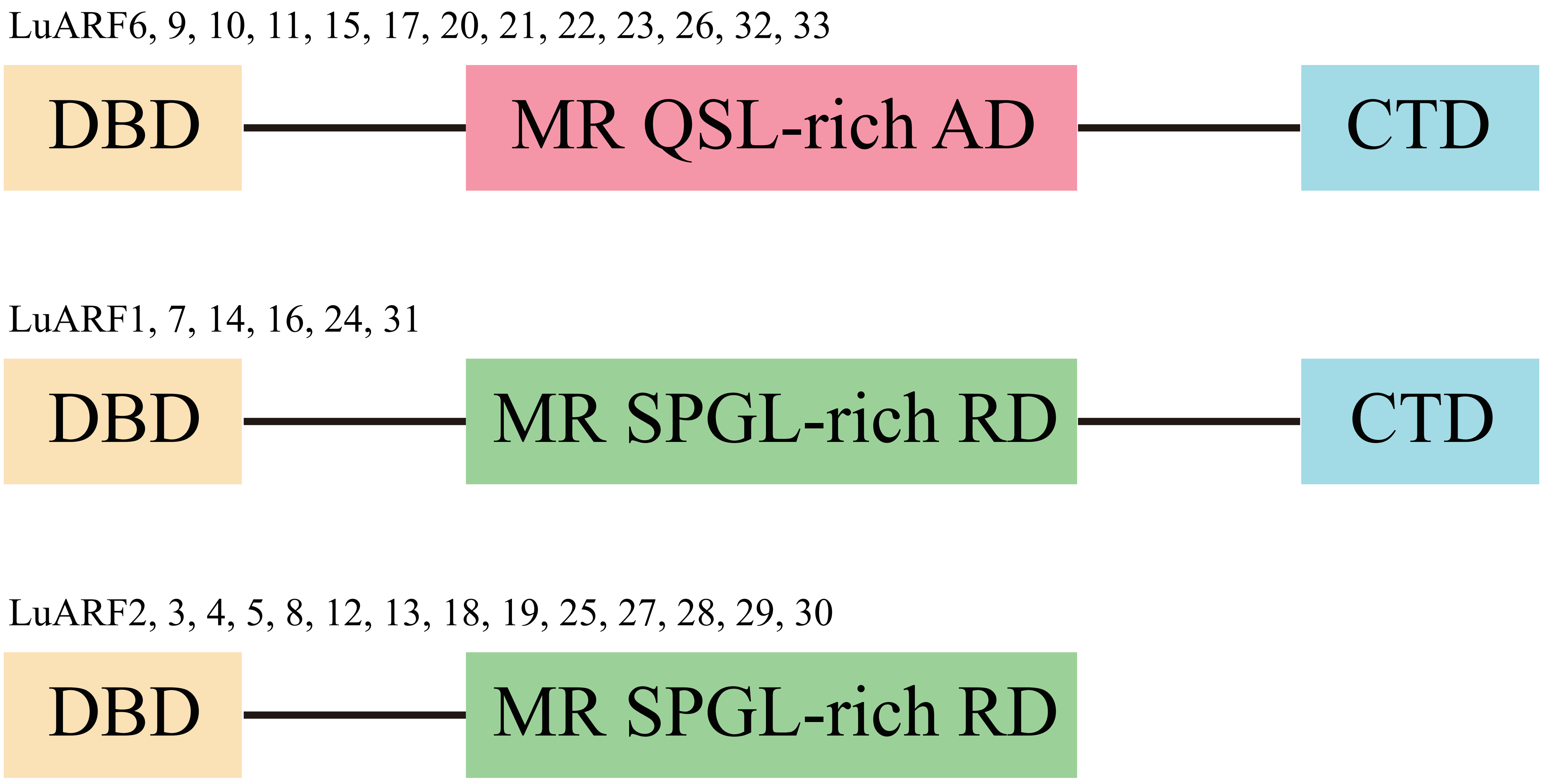

Figure S2. The protein structure of LuARF family.

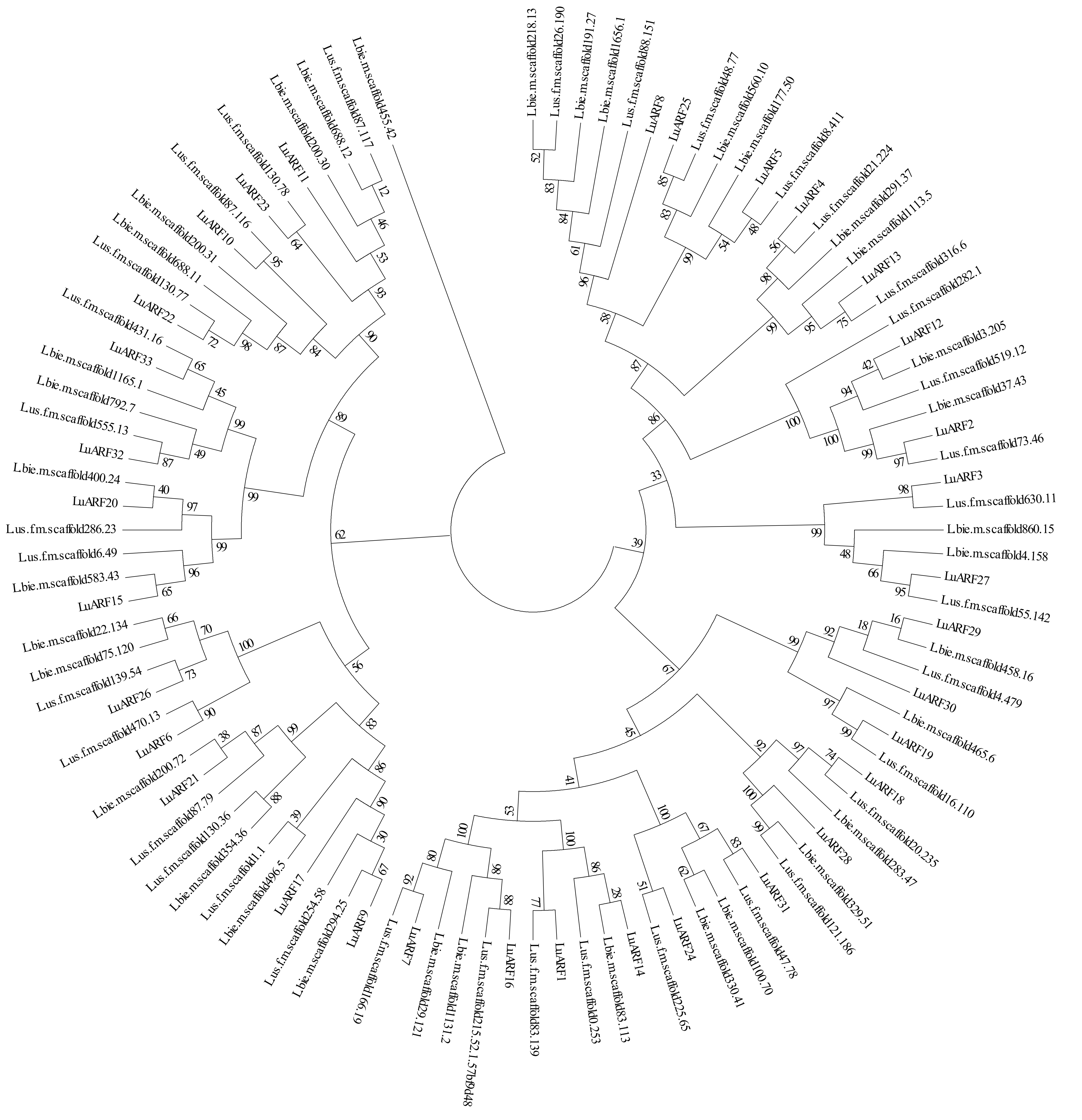

Figure S3. Phylogenetic tree of ARF proteins from Longya-10, Heiya-14 and pale flax.

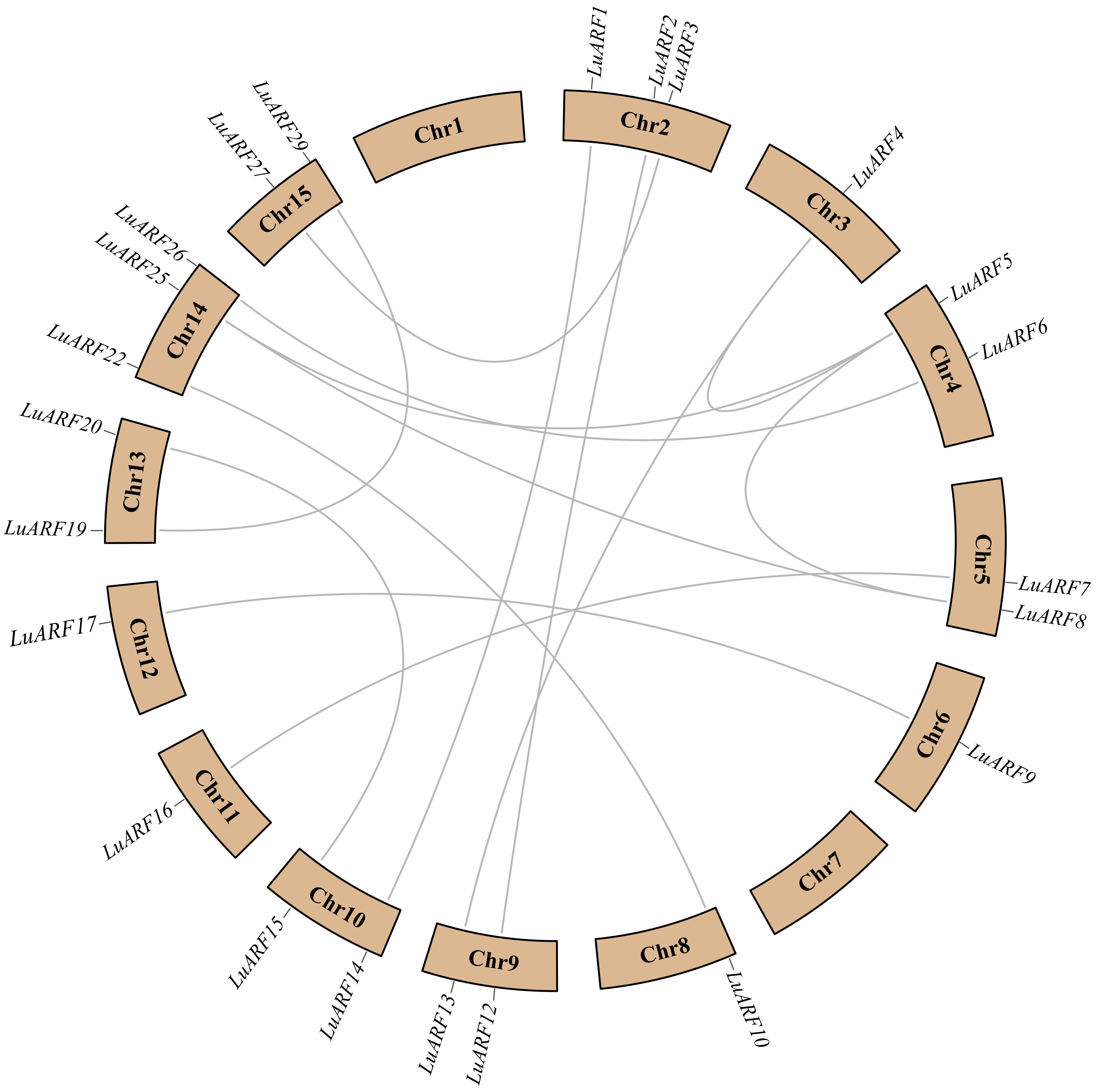

Figure S4. Collinearity of *LuARF* genes in flax genome.
